# Genetic diversity of *Bletilla striata* assessed by SCoT and IRAP markers

**DOI:** 10.1186/s41065-018-0074-4

**Published:** 2018-11-12

**Authors:** Yan Guo, Lina Zhai, Hao Long, Nipi Chen, Chengxian Gao, Zhishan Ding, Bo Jin

**Affiliations:** 1 College of Life Science, Zhejiang Chinese Medical University, Hangzhou, 310053 China; 2 College of Medical Technology, Zhejiang Chinese Medical University, Hangzhou, 310053 China

**Keywords:** *Bletilla striata*, Genetic diversity, SCoT, IRAP, Cluster analysis

## Abstract

**Background:**

*Bletilla striata* is a well-known traditional Chinese herb with varieties of functions. In China, the natural resources of *Bletilla striata* have been severely damaged because of the excessive exploitation and destruction of natural habitats. The aim of present study was to provide a reference for fully exploiting and utilizing the germplasm resources of *Bletilla striata*.

**Results:**

The genetic diversity of 50 varieties of *Bletilla striata* from different area in China was analyzed by SCoT and IRAP molecular marker technique. A total of 209 bands were amplified by 20 groups of SCoT primers, of which 201 (96.17%) were polymorphic, and 47 polymorphic bands (94%) were observed in 50 bands amplified by 8 groups of IRAP primers. The 50 populations of *Bletilla striata* were divided into two major groups by SCoT and IRAP at the genetic similarity coefficient value of 0.60 and 0.68 individually. The partition of clusters in the unweighted pair group method with arithmetic mean dendrogram and principal coordinate analysis plot based on the SCoT and IRAP markers was similar.

**Conclusions:**

Results indicated the abundant genetic diversity of *Bletilla striata* among different areas. Our results will provide useful information for resource protection.

## Background

*Bletilla striata* is a well-known traditional Chinese herb, which was first described in Shennong BenCao Jing (Shennong’s Materia Medica) 2000 years ago. In China, there are four unique populations: *Bletilla striata*, *Bletilla formosana*, *Bletilla ochracea* and *Bletilla sinensis* [20]. Traditional Chinese Medicine holds that *Bletilla striata* is capable of restraining blood leakage, stopping bleeding, dispersing swelling and promoting tissue regeneration. Thus it could be effectively applied in the treatment of hematemesis, hemoptysis, traumatic bleeding, chapped kin, and ulcerative carbuncle [4, 17]. Besides, *Bletilla striata* can be added in medicated diets or drinks when stewed together with chicken or duck and extracted by boiling water or brewed as wine material [14]. Additionally, the non-medical uses of *Bletilla striata* include rubbing its mucilaginous roots in inkstones with vermilion for writing [7], and another use is as an insecticide [12]. In industry, Bletilla extract is used as a coating agent and cosmetic additive [14]. Moreover, the plant also has very high decorative value [14]. The wide uses of *Bletilla striata* results in demand exceeding supply.

In China, the natural resources of *Bletilla striata* have been severely damaged because of the excessive exploitation and destruction of natural habitats [14]*.* More than 10 years ago, *Bletilla striata* was listed as one of the key protected wild medicinal plants (http://rep.iplant.cn/). The price of *Bletilla striata* has soared 20-fold in the past 10 years [14]. In order to solve the problem of *Bletilla striata* resources, the artificial cultivation of *Bletilla striata* and its related species has been developed in the majority of regions in China [9]. More importantly, research on strengthening the selection and breeding of excellent varieties of *Bletilla striata* also has been launched [22]. As part of the efforts to protect the precious plant and explore the best way to utilize the entire plant, our laboratory established good practice for the genetic relationships of *Bletilla* until now.

Molecular markers are one of the most important methods to allow cultivar identification. They are widespread used on account of their simple operation and high detection efficiency [8]. The different DNA-based molecular markers unfold plant variability directly at genetic levels with reliable data required for the estimation of genetic diversity [2], such as inter-simple sequence repeats (ISSR), inter-retrotransposon amplified polymorphism (IRAP), or start codon targeted (SCoT) polymorphism [13]. In this investigation, SCoT and IRAP were used to assess genetic constancy of *Bletilla striata.*

The objectives of this study are to investigate the genetic relationships among some *Bletilla striata* species in China and construct a molecular phylogenetic tree, which provides a theoretical basis for protection, selection and development.

## Results

The oligo nucleotide sequences of SCoT and IRAP primers are summarized in Table 1.

**Table 1:** All molecular marker primers sequence of SCoT and IRAP used in this research

SCoT primer				IRAP primer	
Code	Sequence(5’to3’)	Code	Sequence(5’to3’)	Code	Sequence(5’to3’)
SC3	CAACAATGGCTACCACCG	SC19	ACCATGGCTACCACCGGC	LIRE3-F	CCTCAAAGCTCTCTTCTCCTTC
SC4	CAACAATGGCTACCACCT	SC20	ACCATGGCTACCACCGCG	LIRE3-R	CCTAGAGTTGTACATTTACATT
SC5	CAACAATGGCTACCACGA	SC21	ACGACATGGCGACCCACA	LIRE-Tar-F	CATYATCRGTGGAGCTCT
SC6	CAACAATGGCTACCACGC	SC22	AACCATGGCTACCACCAC	LIRE-Tar-R	AATCATYYYGAGAGCTCTCC
SC7	CAACAATGGCTACCACGG	SC23	CACCATGGCTACCACCAG	LIRE-del-F1	TTGAAACCACYAGCTCAAGGTA
SC9	CAACAATGGCTACCAGCA	SC24	CACCATGGCTACCACCAT	LIRE-del-F2	TATAAAATGTCRGGTCGTGA
SC10	CAACAATGGCTACCAGCC	SC25	ACCATGGCTACCACCGGG	LIRE-del-R	TCATGRAGRTGATARAGWYTAACC
SC11	AAGCAATGGCTACCACCA	SC26	ACCATGGCTACCACCGTC	DO-F	TCCACAAGGCTATCTATG
SC12	ACGACATGGCGACCAACG	SC27	ACCATGGCTACCACCGTG	DO-R	GAGGGATTGGACTTACTG
SC13	ACGACATGGCGACCATCG	SC28	CCATGGCTACCACCGCCA		
SC14	ACGACATGGCGACCACGC	SC30	CCATGGCTACCACCGGCG		
SC15	ACGACATGGCGACCGCGA	SC31	CCATGGCTACCACCGCCT		
SC16	ACCATGGCTACCACCGAC	SC32	CCATGGCTACCACCGCAC		
SC17	ACCATGGCTACCACCGAG	SC33	CCATGGCTACCACCGCAG		
SC18	ACCATGGCTACCACCGCC	SC34	ACCATGGCTACCACCGCA		

### Genetic diversity revealed by SCoT polymorphic markers

**Fig. 1:**
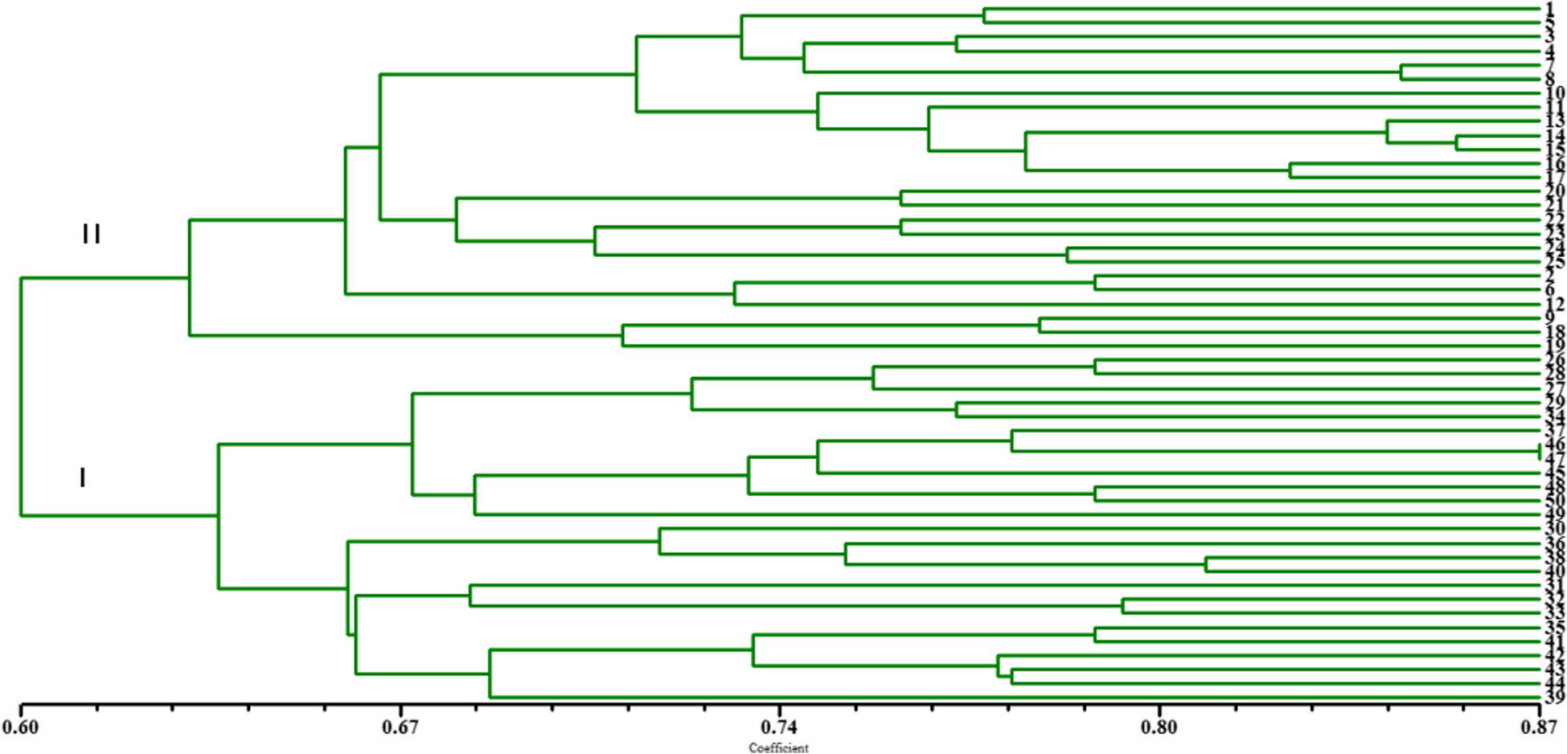
UPGMA dendrogram based on SCoT data showing genetic relationship among the 50 *Bletilla striata*

**Fig. 2:**
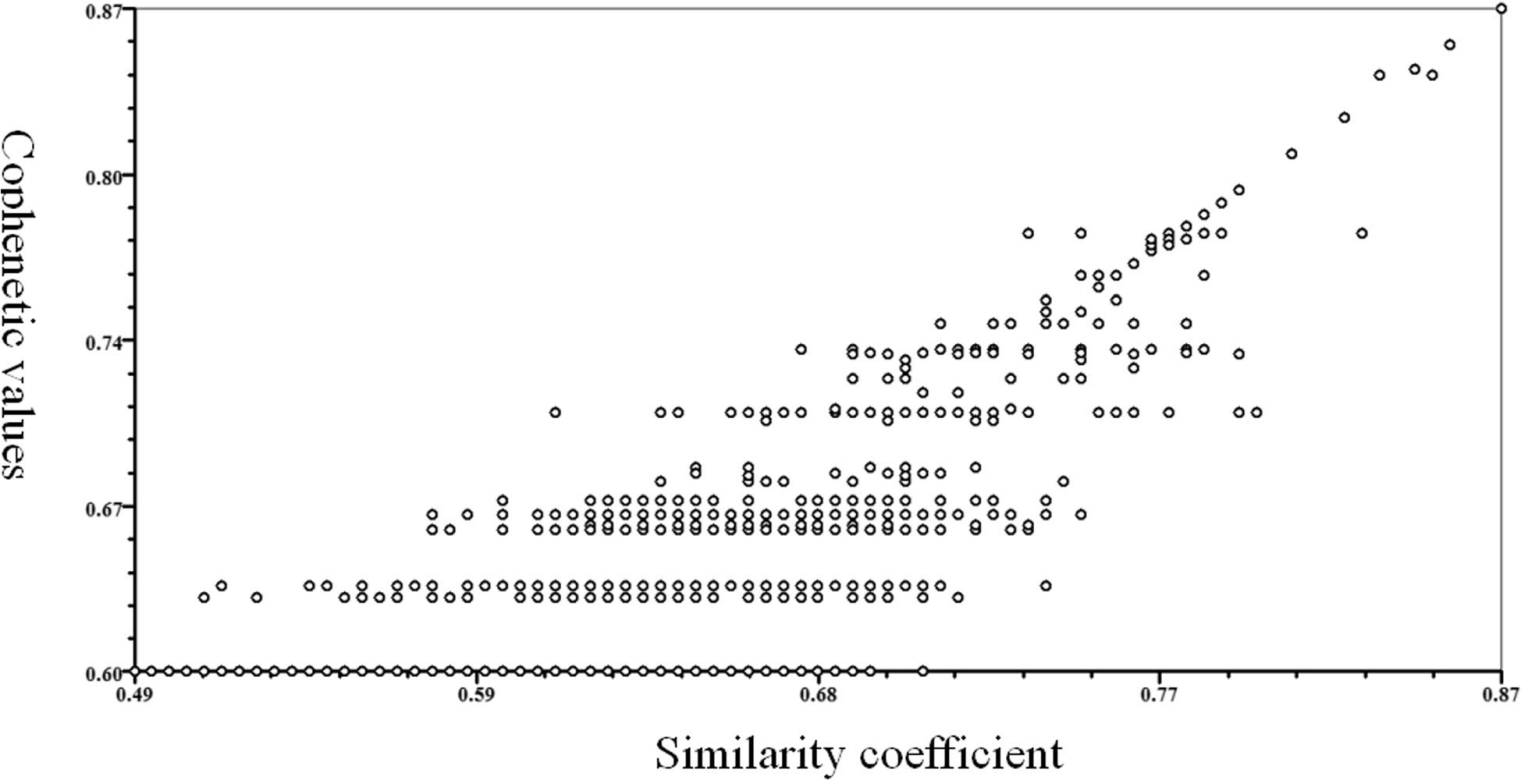
The results of cophenetic correlation analysis among 50 *Bletilla striata* accessions based on SCoT markers

Twenty groups of primers were used to detect genetic polymorphisms in the samples. The primers produced 209 clear SCoT bands, including 201 polymorphism bands. The total number of scored bands varied from 6 (for SC8 + SC11) to 14 (for SC16 + SC21, SC17 + SC20) with an average of 10.5 bands per primer (Table 2). The number of the polymorphic bands ranging from 6 (for SC8 + SC11) to 13 (for SC16 + SC21, SC17 + SC20, SC14 + SC23) with a mean of 96.17% was polymorphic bands per primer (Table 2). The PIC value was ranging from 0.92 to 0.99, with a mean value of 0.96, indicated that the SCoT primers used in the study were effective and polymorphic (Table 2). The Nei’s index varied from 0.28 (for SC18 + SC19) to 0.42 (for SC14 + SC33) with a mean value of 0.37 (Table 2). A dendrogram (Fig. 1) was constructed based on the similarity matrix (data not shown). The highest genetic similarity coefficient (0.87) was found between 38 and 39. With a genetic similarity coefficient value of 0.60, the 50 varieties were divided into two major groups. Group I included twenty-five varieties; the all other varieties were classified into Group II. And the results of cophenetic correlation analysis are shown below (Fig. 2) (the correlation coefficient is *r* = 0.752, indicating that the clustering results are good).

**Table 2:** Characteristics of SCoT and IRAP banding profiles produced in the studied *Bletilla striata*

Primer name	TB	PB	PP(%)	PIC	Nei’s	Fst
IRAP						
Do-F + tar-R	7	5	71.43	0.87	0.45	0.009
DO-F + LIRE-del-F2	5	5	100	0.94	0.40	0.018
DO-F + LIRE-del-F1	9	8	88.89	0.96	0.23	0.012
LIRE-del-F1 + LIRE-Tar-R	7	7	100	0.98	0.35	0.023
LIRE3-R + LIRE-del-R	6	6	100	0.95	0.25	0.011
LIRE3-R + LIRE-TAR-R	6	6	100	0.97	0.36	0.023
LIRE3R + DO-R	6	6	100	0.91	0.38	0.019
LIRE-Tar-F + LIRE-del-F1	4	4	100	0.85	0.42	0.012
Total						
Mean	50	47				
SCOT	6.25	5.88	95.04	0.93	0.36	0.016
SC17 + SC30	10	10	100	0.98	0.36	0.007
SC5 + SC14	11	11	100	0.97	0.42	0.009
SC8 + SC11	6	6	100	0.92	0.39	0.012
SC14 + SC33	10	9	90	0.93	0.42	0.016
SC15 + SC32	11	11	100	0.97	0.33	0.016
SC16 + SC21	14	13	92.86	0.96	0.41	0.015
SC17 + SC20	14	13	92.86	0.92	0.33	0.013
SC18 + SC19	12	11	91.67	0.97	0.28	0.017
SC3 + SC34	7	7	100	0.94	0.34	0.021
SC4 + SC33	9	8	88.89	0.94	0.39	0.023
SC5 + SC32	10	9	90	0.97	0.38	0.017
SC6 + SC31	10	10	100	0.98	0.35	0.019
SC7 + SC30	9	9	100	0.98	0.41	0.017
SC9 + SC28	10	9	90	0.98	0.33	0.015
SC10 + SC27	12	12	100	0.96	0.39	0.017
SC11 + SC26	10	10	100	0.96	0.29	0.016
SC12 + SC25	12	11	91.67	0.95	0.38	0.019
SC13 + SC24	8	8	100	0.99	0.37	0.021
SC14 + SC23	13	13	100	0.99	0.34	0.023
SC15 + SC22	11	11	100	0.93	0.36	0.021
Total	209	201				
Mean	10.45	10.05	96.40	0.96	0.37	0.017

*Abbreviation*: *TB* Total bands, *PB* Polymorphic bands, *pp* Polymorphic percentage, *PIC* Polymorphic Information Content, *Fst* Differentiation between populations index

### Genetic diversity revealed by IRAP polymorphic markers

**Fig. 3:**
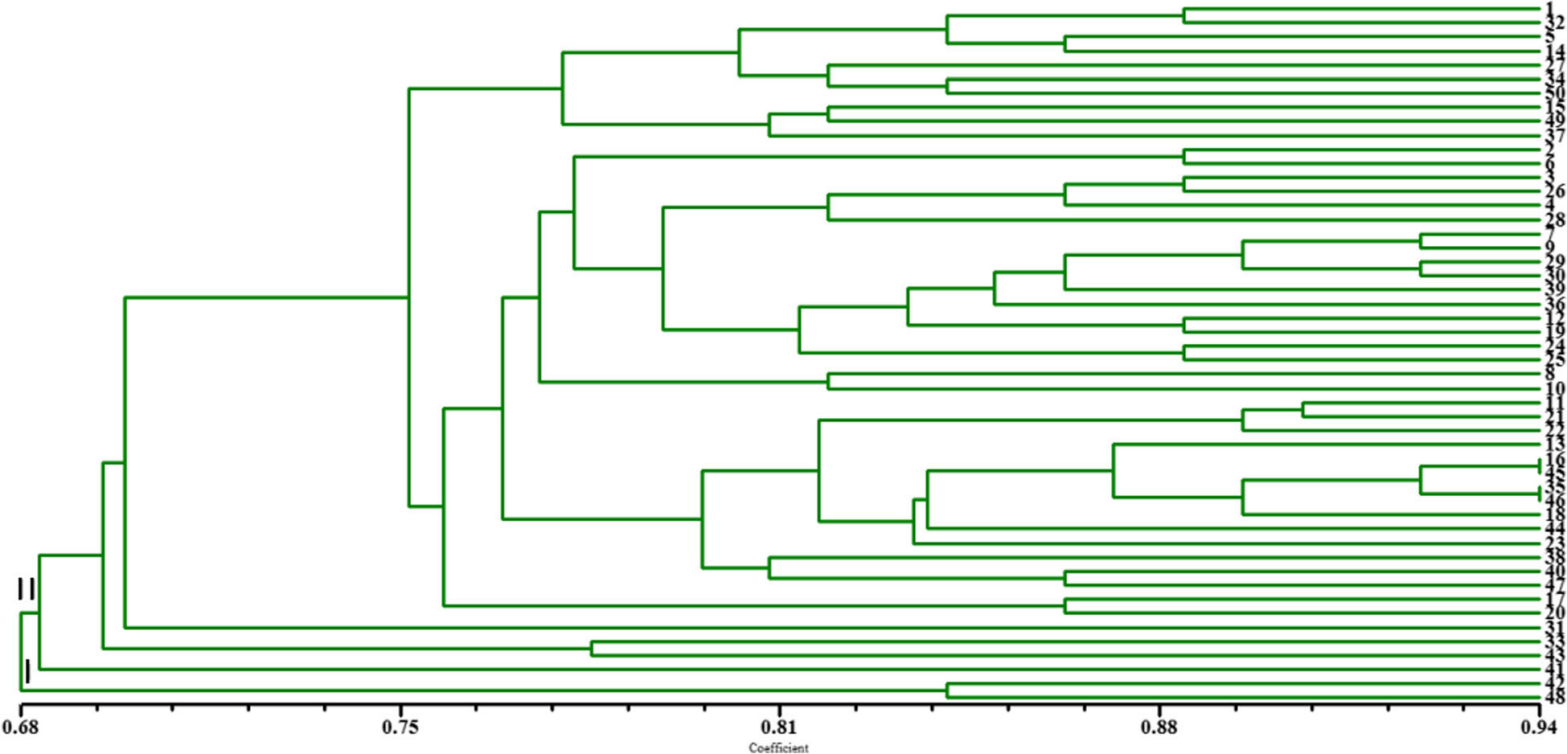
UPGMA dendrogram based on IRAP data showing genetic relationship among the 50 *Bletilla striata*

**Fig. 4:**
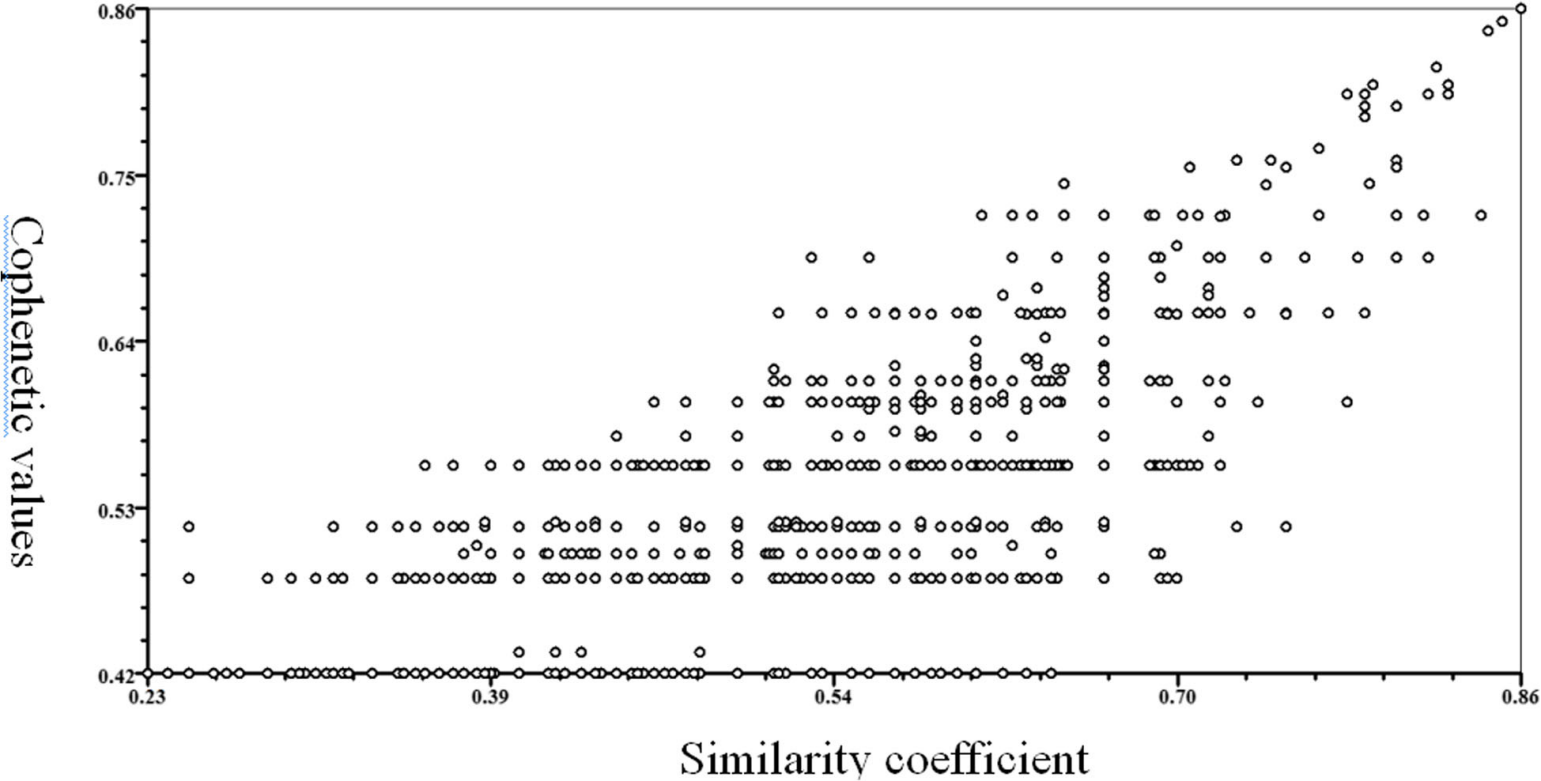
The results of cophenetic correlation analysis among 50 *Bletilla striata* accessions based on IRAP markers

Eight groups of primers were used to detect genetic polymorphisms in the samples. The primers produced 50 clear IRAP bands, including 47 polymorphism bands. The total number of scored bands varied from 4 (for LIRE-Tar-F + LIRE-del-F1) to 9 (for DO-F + LIRE-del-F1) with an average of 6.3 bands per primer (Table 2). The number of the polymorphic bands ranging from 4 (for LIRE-Tar-F + LIRE-del-F1) to 8 (for DO-F + LIRE-del-F1) with a mean of 94% was polymorphic bands per primer (Table 2). The PIC value was ranging from 0.85 to 0.98, with a mean value of 0.93, indicated that the IRAP primers used in the study were effective and polymorphic (Table 2). The Nei’s index varied from 0.23 (for DO-F + LIRE-del-F1) to 0.43 (for Do-F + tar-R) with a mean value of 0.36 (Table 2). A dendrogram (Fig. 3) was constructed based on the similarity matrix (data not shown). The highest genetic similarity coefficient (0.94) was found between 12, 26, 32 and 38. With a genetic similarity coefficient value of 0.78, the 50 varieties were divided into two major groups. Group I included two varieties: 19 and 42; the all other varieties were classified into Group II. And the results of cophenetic correlation analysis are shown below (Fig. 4) (the correlation coefficient is *r* = 0.707, indicating that the clustering results are good).

### Analysis of PCoA

**Fig. 5:**
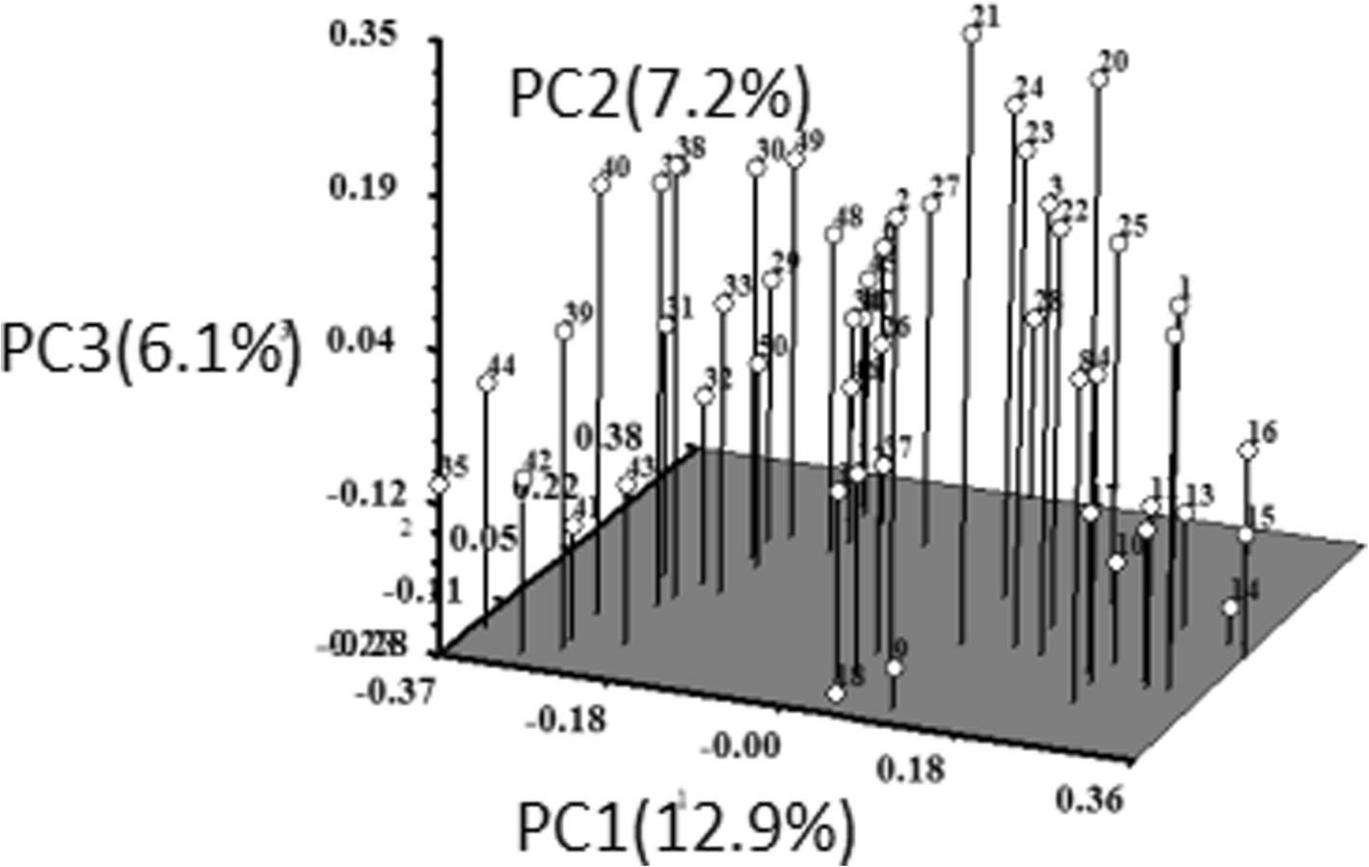
Three-dimensional plots of PCoA indicating relationships among 50 *Bletilla striata* accessions based on SCoT markers

**Fig. 6:**
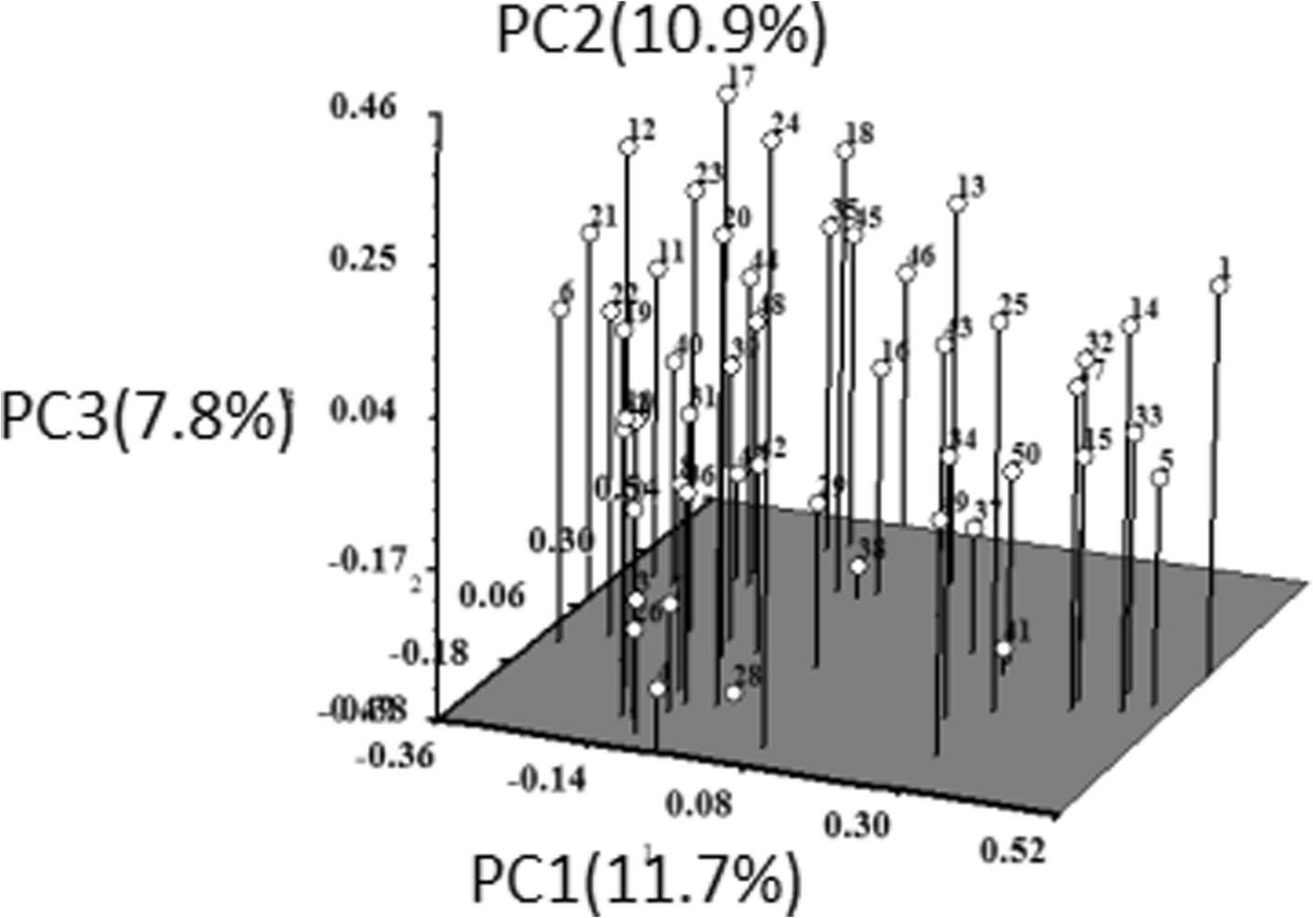
Three-dimensional plots of PCoA indicating relationships among 50 *Bletilla striata* accessions based on IRAP markers

PCoA analysis was done to see the displacement of the accessions and to further confirm the clustering pattern obtained from the dendrogram. The result of the three-dimensional (3D) plots of PCoA analysis for SCoT marker analysis was also similar to that of clustering in the dendrogram (Fig. 5). PCoA analysis indicated the first three axes accounted for 26.2% of total variation among all the accessions studied. The first, second and third axes represented 12.9, 7.2 and 6.1% of the variation respectively. The result of the three-dimensional (3D) plots of PCoA analysis for IRAP marker analysis was similar to that of clustering in the dendrogram (Fig. 6). PCoA analysis indicated the first three axes accounted for 30.4% of total variation among all the accessions studied. The first, second and third axes represented 11.7, 10.9 and 7.8% of the variation respectively.

### Analysis of molecular variance (AMOVA)

The analysis of molecular variance (AMOVA) of the SCoT data revealed that out of the total recorded variation, 79.38% was recorded within the species level whereas 20.62% was compartmentalized amongst the species. Similar in distribution to that of SCoT, IRAP marker data accounted for 77.36% variability at inter-specific level and 22.64% at intra-specific level. Which indicated high genetic variation within populations, and using SCoT marker reveled higher variation within and among populations of *Bletilla striata* (Table 3)*.*

**Table 3:** Analysis of molecular variance (AMOVA) among and within species based on SCoT marker (a) and IRAP marker (b)

Source of variation	df	Sum of squares	Variance components	Variation (%)	*P*-value
a					
Among populations	3	385.11	1.87054	20.62	*P* < 0.001
Within populations	46	783.35	21.3307	79.38	P < 0.001
Total	49	2049.35	23.52211		
b					
Among populations	3	365.49	2.66702	22.64	P < 0.001
Within populations	46	667.90	27.3087	77.36	P < 0.001
Total	49	1329.72	30.41602		

## Discussion

In general, the genetic characteristics of a species are affected primarily by various ecological and biological traits, modes of reproduction and breeding and also by the various human mediated anthropogenic activities (Hamrick and Godt 1996; Nybom 2004). The genetic background of wild *Bletilla striata* becomes complex because of prolonged cross pollination and natural selection. In this study, we collected a variety of *Bletilla striata* from China. The genetic diversity and phylogenetic relationships of the samples were analyzed using SCoT and IRAP markers. The SCoT marker systems are proved to be efficient and inexpensive ways to provide molecular data to assess genetic diversity, and it has been used successfully to determine genetic relationships for many plants ([15]; Bhattacharyya et al. 2013; [21]) whereby primers target the short-conserved region flanking the ATG translation start codon of plant genes [5]. IRAP is developed by Kalendar et al. [6] and initially designed to identify different barley (*H. vulgare*) cultivars. IRAP markers involve the PCR amplification of DNA sequences between two nearby retrotransposons, using a primer designed from the LTR sequence of a retrotransposon, which were recently used extensively in studying genetic diversity, genetic relationship and germplasm management [15, 21]. In the ATG flanking region, 20 groups of SCoT primers obtained a ratio of 96.17% polymorphism. In the LTR region, 8 groups of IRAP primer combinations of polymorphic fragments obtained a ratio of 94%. The results indicated that both the SCoT and IRAP markers are equally efficient in the detection of polymorphism. However, SCoT proved to have slightly higher detection capacity i.e. in comparison to IRAP.

Sometimes, making use of only one marker for evaluating the genetic diversities among species may ignore a lot of unanswered questions [18]. However, combining polymorphic information derived from different marker systems was expected to decrease the effects of their independent inaccuracies [11]. Genetic relationship evaluation using a combination of SCoT+IRAP would result in a more accurate and reliable analysis of genetic diversities compared with a single marker. Our report is the first to integrate SCoT and IRAP data to elucidate the genetic relationships among Bletilla species, which can serve as a basis for the establishment of an effective system for species identification and genetic diversity analysis of *Bletilla striata*.

The UPGMA dendrograms indicated the abundant genetic diversity of the samples. *Bletilla striata* collected from one province can be clustered into nearly 2 clusters. The genetic distances of *Bletilla striata* from different provinces can be very close regardless of the flowers’ color. Therefore, combining the current status of online trade prevalence, we suspect that exchanging universally results in the serious situation of transplanting and cross planting, which may cause high genetic variation among the populations from different areas. At the same time, we found that *Bletilla striata* collected from the same province are also far apart. The reason for this separation can be its growth background. *Bletilla striata* from different region may change the genetic information due to the hybridization between different parental, with the coaction of the effect of the soil, nutrients, temperature, and moisture resulting in differences in genetic traits, making the genetic diversity of *Bletilla striata* from the same area more complicated. Furthermore, the UPGMA clustering indicated that there was no significant relationship between genetic and geographic distances. This result was confirmed by the PCoA which indicates that the geographical distribution is not the main factor that shaped the current population genetic structure. For those populations with high levels of genetic variation of different regions, we suggest that their habitats should be protected and the exploitation of wild resources be controlled.

Experiments have shown that the genetic relationships among different geographical origins of *Bletilla striata* are complex. As a valuable Chinese herbal medicine, to protect and take artificial breeding of wild resources are very important. We hope that this research provides some references and theoretical basis for the identification and selection of good seeds of *Bletilla striata* resources.

## Conclusions

The primary objective in nature conservation is to preserve the evolutionary potential of species through maintaining as much genetic diversity as possible. Thus, knowledge of the genetic variation between and within different populations of plant species plays a significant role in the formulation of appropriate strategies for their conservation [10]. In summary, our study revealed that 50 samples of *Bletilla striata* depicted a high level of genetic diversity. SCoT and IRAP markers can provide highly accurate and efficient information on population genetic diversity. This study provides valuable baseline data on the population genetics of *Bletilla striata* from China, which is useful for germplasm appraisal and resource protection, including the construction of a core collection and regional variety distribution and subrogation. Finally, it is noted that the agarose gel electrophoresis detection is simple and easy to operate, but it is often difficult to get a clear, easily distinguishable photo. Even a large number of repeated tests are required to meet the final test requirements. Capillary electrophoresis may be used in the future to obtain more accurate analysis results.

## Material and methods

### Materials

**Fig. 7:**
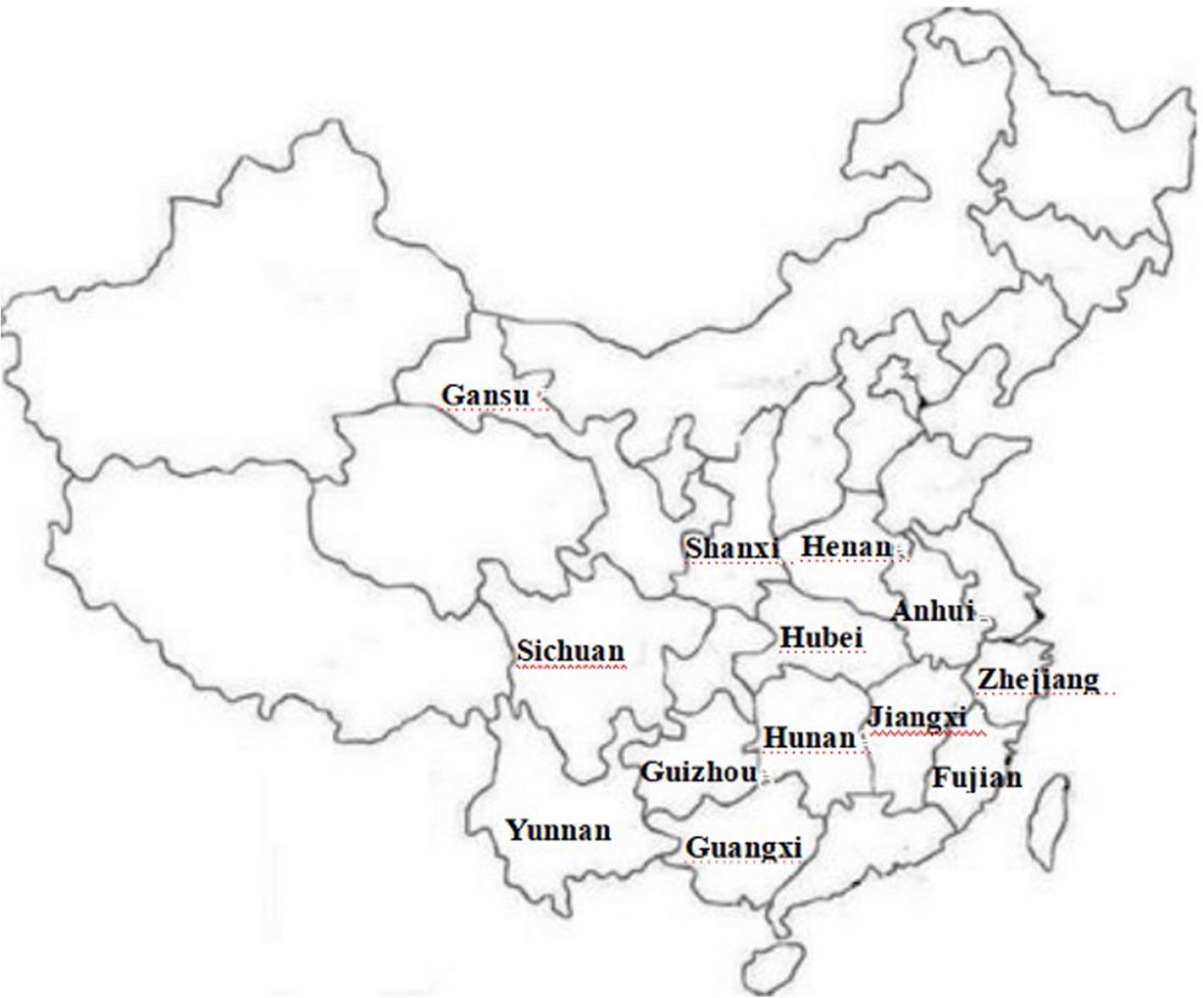
Collecting sites for the *Bletilla striata* species in China are marked

The *Bletilla striata* collected in this experiment were identified by professor Zhishan Ding of Zhejiang Chinese Medical University. A voucher specimen of the plant material used in this study has been deposited in molecular biology laboratory of Zhejiang Chinese Medical University (From NO.BJ-20160301-001 to NO.BJ-20160301-050). Specific information is in Table 4, Fig. 7.

**Table 4:** Sources and description of materials

No	Location	Species name	Flower color
1	Hubei Province, China	*Bletilla striata*	Purple
2	Hubei Province, China	*B1etilla sinensis*	White
3	Hubei Province, China	*Bletilla striata*	Purple
4	Hubei Province, China	*Bletilla ochracea*	Yellow
5	Hubei Province, China	*Bletilla striata*	Purple
6	Hubei Province, China	*Bletilla striata*	Purple
7	Hubei Province, China	*Bletilla striata*	Purple
8	Hubei Province, China	*Bletilla striata*	Purple
9	Hubei Province, China	*B1etilla sinensis*	White
10	Hubei Province, China	*Bletilla striata*	Purple
11	Hubei Province, China	*Bletilla striata*	Purple
12	Hubei Province, China	*B1etilla sinensis*	White
13	Hubei Province, China	*Bletilla striata*	Purple
14	Hubei Province, China	*Bletilla striata*	Purple
15	Anhui Province, China	*Bletilla striata*	Purple
16	Anhui Province, China	*Bletilla striata*	Purple
17	Anhui Province, China	*Bletilla striata*	Purple
18	Anhui Province, China	*Bletilla striata*	Purple
19	Anhui Province, China	*Bletilla striata*	Purple
20	Anhui Province, China	*Bletilla striata*	Purple
21	Sichuan Province, China	*Bletilla striata*	Purple
22	Sichuan Province, China	*Bletilla striata*	Purple
23	Sichuan Province, China	*Bletilla striata*	Purple
24	Sichuan Province, China	*B1etilla sinensis*	White
25	Henan Province, China	*Bletilla striata*	Purple
26	Henan Province, China	*Bletilla striata*	Purple
27	Henan Province, China	*Bletilla striata*	Purple
28	Guizhou Province, China	*Bletilla striata*	Purple
29	Guizhou Province, China	*Bletilla striata*	Purple
30	Yunnan Province, China	*Bletilla striata*	Purple
31	Yunnan Province, China	*Bletilla striata*	Purple
32	Yunnan Province, China	*Bletilla striata*	Purple
33	Zhejiang Province, China	*Bletilla striata*	Purple
34	Zhejiang Province, China	*Bletilla striata*	Purple
35	Zhejiang Province, China	*Bletilla striata*	Purple
36	Zhejiang Province, China	*Bletilla striata*	Purple
37	Zhejiang Province, China	*Bletilla striata*	Purple
38	Zhejiang Province, China	*Bletilla striata*	Purple
39	Zhejiang Province, China	*Bletilla striata*	Purple
40	Shanxi Province, China	*Bletilla striata*	Purple
41	Shanxi Province, China	*B1etilla sinensis*	White
42	Shanxi Province, China	*Bletilla striata*	Purple
43	Shanxi Province, China	*Bletilla striata*	Purple
44	Shanxi Province, China	*Bletilla striata*	Purple
45	Gansu Province, China	*Bletilla striata*	Purple
46	Hunan Province, China	*Bletilla striata*	Purple
47	Fujian Province, China	*Bletilla striata*	Purple
48	Jiangxi Province, China	*Bletilla striata*	Purple
49	Jiangxi Province, China	*Bletilla striata*	Purple
50	Guangxi Province, China	*Bletilla striata*	Purple

### Leaf DNA extraction

A modified cetyltrimethyl ammonium bromide (CTAB) method was used for DNA extraction [21]. DNA samples were stored at − 20 °C and the quality verified by electrophoresis on ethidium bromide stained 1% agarose gel.

### Determination of IRAP and SCoT systems

Karim Sorkheh et al. used IRAP for germplasm analysis of wild *Pistacia chinensis* [16], and Ahmad Mousapour Gorji et al. used SCoT to analyze the genetic fingerprint of tetraploid potatoes [1]. We used 20 μL of the IRAP and SCoT molecular markers in both studies.

### SCoT analysis

PCR amplification was performed in a final reaction volume of 20 μL containing 10× buffer, 0.1 mM dNTPs, 0.3 μM of each primer, 1 U Taq polymerase, and 20 ng of template DNA. The amplification was performed with the following thermal program: 94 °C for 4 min; followed by 36 cycles of denaturation at 95 °C for 50s, annealing at 50 °C for 40 s, elongation at 72 °C for 2 min; and extension at 72 °C for 5 min. All PCR reactions were performed twice. The products were kept at 4 °C.

### IRAP analysis

PCR amplification was performed in a final reaction volume of 20 μL containing 10× buffer, 800 mM dNTPs, 0.3 nM of each primer, 1.25 U Taq polymerase, and 20 ng of template DNA. The amplification was performed with the following thermal program: 94 °C for 4 min, 36 °C for 1 min, 72 °C for2 min; followed by 40 cycles of denaturation at 94 °C for 1 min, annealing at 36 °C for 1 min, elongation at 72 °C for 2 min; and extension at 94 °C for 4 min, 36 °C for 1 min, 72 °C for2 min. All PCR reactions were performed twice. The products were kept at 4 °C.

### Electrophoresis detection and genotypes of reaction products

DNA amplification products for SCoT and IRAP markers were analyzed by electrophoresis in 1.80% agarose gels. The electrophoretic patterns of the products were recorded using a gel imaging system (Tanon 3500R, Shanghai, China). Genotypes were built based on different DNA band patterns on gels.

### Data analyses

Data matrices of Inter-simple sequence repeat and start codon targeted polymorphic marker profiles were generated by scoring (1) for presence and (0) for absence of individual allele. Polymorphism information content (PIC) is a property value of a marker based on its allelic number and distribution frequency in a population. PIC for marker *ί* was calculated using PIC = 1 − Σ*Pί*^2^ according to Botstein et al. [3], where *Pί* is the allele frequency at locus *ί*. Nei’s genetic diversity index (ℎ) was calculated using POPGENE 1.31[19]. The resulting matrices were used to construct the dendrogram through unweighted pair-group method with arithmetic mean (UPGMA) using SAHN module. The cluster, cophenetic correlation and PCoA analysis was conducted by NTSYSpc software version 2.1. Analysis of molecular variance (AMOVA) was performed in order to determine the differences existent amongst and within population levels. F-statistics (Fst) was also calculated using Arlequin v. 3.01 (Miller 1998).
